# Moderating Effect of the Lean Tissue Index on the Relationship between the Trabecular Bone Score and Augmentation Index in Dialysis Naïve Patients with Stage 5 Chronic Kidney Disease

**DOI:** 10.3390/jcm11133897

**Published:** 2022-07-04

**Authors:** Byoung-Geun Han, Daewoo Pak, Jun Young Lee, Jae-Seok Kim, Jae-Won Yang, Ki-Youn Kwon

**Affiliations:** 1 Department of Nephrology, Yonsei University Wonju College of Medicine, Wonju 26426, Korea; neptune@yonsei.ac.kr (B.-G.H.); junyoung07@yonsei.ac.kr (J.Y.L.); ripplesong@hanmail.net (J.-S.K.); kidney74@yonsei.ac.kr (J.-W.Y.); 2 Division of Data Science, Yonsei University, Wonju 26493, Korea; dpak@yonsei.ac.kr; 3 Department of Orthopedic Surgery, Yonsei University Wonju College of Medicine, Wonju 26426, Korea

**Keywords:** body composition, bone mineral density, chronic kidney disease, impedance, trabecular bone, vascular stiffness

## Abstract

Osteopenia, sarcopenia, and increased vascular stiffness are common in patients with chronic kidney disease-mineral bone disorder (CKD-MBD) with protein energy wasting and can lead to worse clinical outcomes. We investigated the potential moderating role of the lean tissue index (LTI) in the relationship between bone microarchitecture and vascular stiffness in dialysis naïve patients with stage 5 CKD. Bioimpedance spectroscopy for evaluating LTI, lumbar spine dual energy X-ray absorptiometry for determining the trabecular bone score (TBS), and arterial applanation tonometry measurements for the central augmentation index, at a heart rate of 75 beats/minute (cAIx75), were simultaneously performed in 117 consecutive patients. A hierarchical regression analysis was conducted to assess the moderating effect of LTI on the relationship between TBS and cAIx75 after adjusting for age and sex. The effect of the interaction between LTI and TBS on cAIx75 was statistically significant (*p* = 0.030), demonstrating that the cAIx75 tends to decrease more, with the joint effect of LTI and TBS. In the separate analyses, the interaction effect was significant only in women (*p* = 0.048) and the group of diabetes (*p* = 0.042). Our study suggests that the evaluation of changes in body composition, bone health, and vascular stiffness needs to be performed simultaneously in patients with advanced-stage CKD. Further research in patients with different stages of CKD warranted to generalize and apply our results to patients in other stages.

## 1. Introduction

Cardiovascular disease is the leading cause of death in patients with stage 4 chronic kidney disease (CKD), regardless of the cause, with an incidence close to 45% [1]. Biomarkers associated with increased cardiovascular risk in CKD include uncontrolled blood pressure, left ventricular mass, carotid-femoral pulse-wave velocity, left ventricular systolic and diastolic function, fluid overload, and vascular calcification (VC). Among these biomarkers, VC is expressed in the general population with increasing age, but in CKD patients, VC can be induced regardless of age, which appears to be one of the various clinical features of chronic kidney disease-mineral bone disorder (CKD-MBD) [2]. The uremic milieu induced by reduced kidney function has been reported as a triggering and accelerating factor for VC [3,4]. Medial calcification of the vessel wall is a hallmark of arteriosclerosis and plays an important role in the development of arterial stiffness in patients with advanced CKD [5]. Notably, VC and vascular stiffness are independent predictors of cardiovascular mortality [6,7].

Some studies have demonstrated a positive relationship between a low bone mineral density (BMD) and the presence of VC [8,9]. A low BMD was also associated with increased cardiovascular disease related mortality [10]. Moreover, a lower bone mass in patients with end-stage renal disease (ESRD) is more closely related to accelerated VC and increased cardiovascular events, than in the general population [11,12,13,14]. Some studies have reported that aortic stiffness was related to patients’ body fat percentage and total lean mass [15,16]. Patients with CKD and sarcopenia had a higher risk of mortality [17]. Therefore, low levels of bone and lean tissue mass can be considered as biomarkers reflecting increased mortality.

Most studies, including the aforementioned studies, have investigated a single relationship between bone mass and VC or vascular stiffness, or have evaluated the degree of influence of a single factor on the outcomes of cardiovascular disease. In addition, several studies have evaluated the simple effect of changes in body composition, such as muscle mass or fat distribution, on vascular stiffness. Osteoporosis, sarcopenia, and vascular disease may share common basic mechanisms, and risk factors in aging [18,19]. Conversely, osteoporosis, muscle dysfunction or sarcopenia, or VC and dysfunction develop individually or in complex patterns, simultaneously, in patients with CKD-MBD with protein energy wasting, regardless of age.

Few studies have statistically analyzed the relationship between bone mass, muscular mass, and vascular stiffness concomitantly in the general population or in a specific group, such as CKD patients. Therefore, we investigated the potential interacting effect of the lean tissue index (LTI) on the relationship between lumbar spine bone microarchitecture and vascular stiffness in dialysis naïve patients with stage 5 CKD (CKD5), who were free of non-traumatic bone fracture.

## 2. Materials and Methods

### 2.1. Patients and Data Collection

Since 2014, we have registered consecutive patients with CKD5, to a bioimpedance cohort, after receiving approval (no. CR319143) from the Institutional Review Board of Yonsei University Wonju Severance Christian Hospital. Of the patient cohorts, 117 patients were simultaneously evaluated for their trabecular bone score (TBS), LTI, and central augmentation index (AIx), at the time of enrollment. All patients underwent the aforementioned tests prior to the initiation of dialysis treatment, as the subjects were hospitalized to plan their first dialysis treatment. Therefore, the current study was a retrospective observational analysis of a prospective cohort database in dialysis naïve patients with CKD5. All patients provided written informed consent prior to their participation in the study. This study was conducted in accordance with the Declaration of Helsinki.

### 2.2. Bone Mineral Density, Body Composition, and Vascular Stiffness Assessment

Areal BMD (aBMD) was measured using a dual-energy X-ray absorptiometry (DEXA) densitometer (Horizon W, Hologic, Inc., Marlborough, MA, USA) at the lumbar spine (L1 to L4) and at femur neck in anterior-posterior projection. TBS was obtained from lumbar spine DEXA 2-D images for the same region of interest as that of the BMD measurement, using TBS iNsight Software^®^ (version 2.1.2.0, Medimaps, Merignac, France). Body composition was defined using a multi-frequency bioimpedance spectroscopy system [Body Composition Monitoring^TM^ (BCM^TM^), Fresenius Medical Care AG and Co., Bad Homburg vor der Höhe, Germany]. BCM^TM^ automatically implements and presents the lean tissue mass (LTM) and LTI values. LTM is one of the three main outputs of the body composition model inside the BCM^TM^. LTI is calculated as LTM/height^2^ (kg/m^2^). The normalization by using height^2^ is performed in accordance with body mass index (BMI) to make it more comparable between subjects of different body sizes. The level of overhydration (OH, L) as a marker of fluid balance can be also calculated. Therefore, the patient’s BMI was recalculated for a corrected BMI (cBMI) by considering fluid overload using the following formula: cBMI (kg/m^2^) = (body weight − OH)/height^2^. Arterial stiffness was assessed by measuring central AIx at a heart rate of 75 beats per minute (cAIx75), using an arterial applanation tonometry method (HEM-9000AI; Omron Matsusaka Co., Ltd., Matsusaka, Japan). The cAIx75 was automatically generated. In our study, all investigators performed the measurements without knowing the purpose of the study or any patient information. All machine measurements were performed as described in previous studies [20,21,22].

### 2.3. Statistical Analysis

Patient characteristics were summarized using means and standard deviations for continuous variables and frequencies and percentages for categorical variables. We divided the patients into three groups according to tertiles based on cAIx75 scores. The group difference of continuous variables was compared using analysis of variance and post-hoc testing with the Bonferroni correction. The group difference of categorical variables was compared using chi-squared tests. A trend in the cAIx75 tertiles was analyzed with the Jonckheere-Terpstra trend test. Pearson correlation coefficients were calculated to evaluate the associations of cAIx75, TBS and LTI with laboratory variables. To examine the moderating effect of LTI on the relationship between TBS and cAIx75, we conducted a hierarchical regression analysis, wherein cAIx75 was a dependent variable, and the demographic factors and the interaction term between TBS and LTI were considered as a block being entered in the step of the regression analysis. The separate analyses were also performed for male and female patients, and patients with and without diabetes.

All analyses were performed using IBM SPSS Statistics software (version 25.0; IBM Corporation, Armonk, NY, USA). Graphs were generated using Prism software (version 5.02; GraphPad Software, San Diego, CA, USA) and Jamovi software (version 2.2.5). *p*-values < 0.05 were considered statistically significant.

## 3. Results

### 3.1. Characteristics of the Study Patients

**Figure 1:**
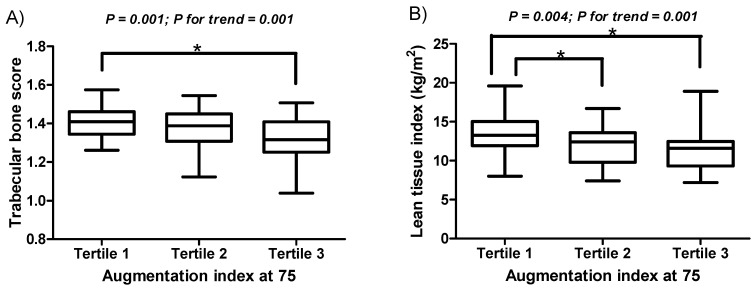
TBS and LTI according to the tertile distribution of augmentation index at 75. There was a decreasing trend in TBS and LTI, with increasing vascular stiffness.

The mean ages of male and female patients were 59.44 ± 12.46 years and 59.31 ± 15.72 years, respectively. Males accounted for 53.8% (*n* = 63) of all patients. Figure 1A TBSs (1.40 ± 0.08 for men and 1.33 ± 0.12 for women; *p* < 0.001) and Figure 1B LTI scores (13.60 ± 2.12 kg/m^2^ for men and 10.74 ± 2.14 kg/m^2^ for women; *p* < 0.001) were significantly higher in men, whereas cAIx75 (70.37 ± 10.97% for men and 84.04 ± 14.45% for women; *p* < 0.001) was significantly higher in women. The cAIx75 tertiles 1, 2, and 3 correspond to <70%, 70–81%, and >81%, respectively. The clinical characteristics of the patients in the three cAIx75 tertiles are presented in Table 1. Patients in the third tertile had significantly lower TBSs, femur neck BMD (FN-BMD), lumbar spine BMD (LS-BMD), height, and LTI values than those in the first tertile. Patients in the third tertile also had a significantly lower lean tissue mass than those in the first tertile. Therefore, we finally decided to use LTI for analysis, as patients’ heights were reflected in LTI. There was a trend towards as lower TBS and LTI across increasing cAIx75 tertiles (Figure 1). Serum biochemical tests showed no significant difference between the three groups. The mean estimated glomerular filtration rate was 6.50 ± 2.33 mL/min/1.73 m^2^.

### 3.2. Correlation of Laboratory Parameters with TBS, LTI, and cAIx75

**Figure 2:**
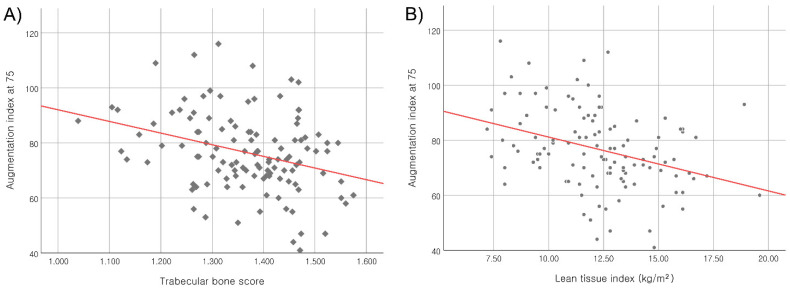
Association between the augmentation index at 75 and (**A**) TBS (*r* = –0.321, *p* < 0.001) and (**B**) LTI (*r* = −0.348, *p* < 0.001).

Both TBS and LTI were negatively associated with cAIx75 (*r* = −0.321, *p* < 0.001 for TBS and *r* = −0.348, *p* < 0.001 for LTI) (Figure 2). Table 2 shows the correlations of the demographic and laboratory parameters with TBS, LTI and cAIx75, respectively. For the demographic parameters, age was positively correlated with cAIx75 and TBS, but not for LTI. Conversely, cBMI was only correlated with TBS. None of the laboratory variables were significantly correlated to cAIx75. Total cholesterol values were positively associated with TBS (*r* = −0.010, *p* = 0.029). For LTI, phosphate levels were significantly correlated (*r* = 0.145, *p* = 0.019), and intact parathyroid hormone (iPTH) and high-sensitivity C reactive protein (hs-CRP) levels were marginally significant (*r* = 0.145, *p* = 0.088 for iPTH and *r* = 0.142, *p* = 0.096 for hs-CRP).

### 3.3. Hierarchical Moderated Regression Analyses

The interaction effect between LTI and TBS was statistically significant after adjusting for age and sex (B = −2.461, t = −2.198, *p* = 0.030) (Table 3). In the analyses performed separately by sex after adjusting for age, the interaction effect was significant only for women (B = −3.738, t = −2.026, *p* = 0.048), which implies that the extent to which cAIx75 decreases is much greater when both TBS and LTI increase (Table 4). In addition, in the comparative analysis between the groups with and without diabetes, the interaction effect of LTI was only found in the group with diabetes (B = −2.436, t = −2.069, *p* = 0.042) after adjusting for age and sex (Table 5).

**Figure 3:**
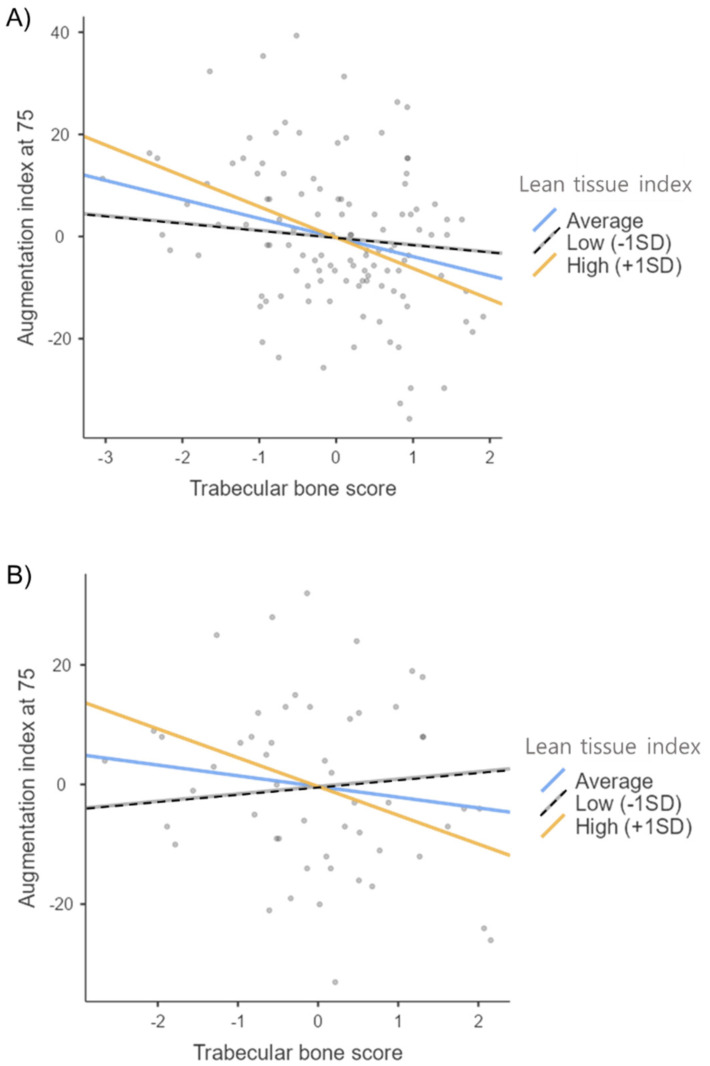

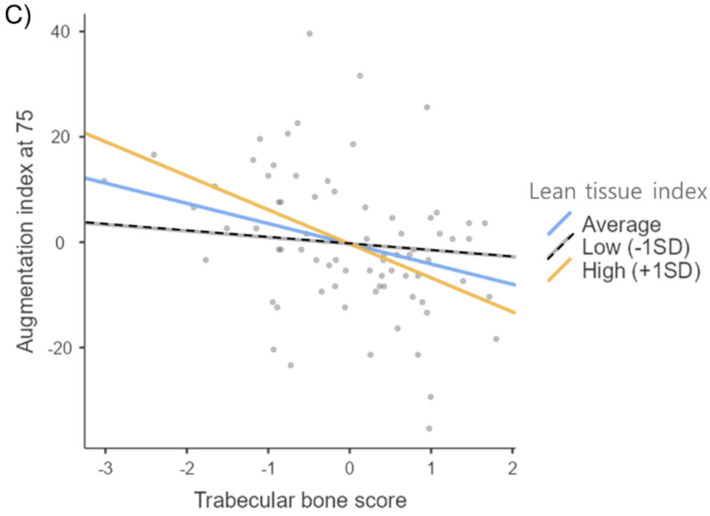
Moderations between TBS and LTI for predicting the augmentation index at 75 separately for (**A**) all patients, (**B**) women, and (**C**) patients with diabetes.

The effect of the interaction of TBS with LTI, on cAIx75 is shown in Figure 3. It demonstrates the predicted values of cAIx75 according to the change in TBS at the average of LTI or at one standard deviation. The cAIx75 tends to decrease as TBS increases with larger LTI values.

## 4. Discussion

CKD-MBD implies that changes occur in the bone and vascular status of CKD patients. In other words, it can be summarized as bone-related VC. VC is a representative cause of vascular stiffness that can be observed in various disease states, such as after menopause or during the aging process [7,9,23]. In particular, VC can be highly observed in CKD, but the mechanism of induction is somewhat different from other diseases [24,25,26,27]. In this study, vascular stiffness was evaluated using cAIx75 derived from pressure waveform analysis. AIx of pressure waveform analysis has been reported as an independent predictor of all-cause and cardiovascular mortality in patients with ESRD [28].

It has been suggested that a low BMD was significantly associated with increased arterial stiffness in previous studies [29,30]. BMD was also associated with increased cardiovascular mortality [10]. Nevertheless, TBS was used for analysis in this study because calcification of the abdominal aorta is a major cause of overestimation of lumbar spine BMD using DEXA [20]. Although TBS provides indirect indices of bone trabecular microarchitecture and bone quality, TBS may be associated with osteoporotic fracture risks independent of BMD in patients with CKD [31]. Trabecular bone loss was associated with VC in CKD [32]. In our study, we did not directly analyze the relationship between TBS and VC, but TBS was independently associated with cAIx75 as a marker of vascular stiffness even after correction for age or sex in multiple regression analyses. CKD has been proposed as a model of early vascular and bone aging.

Body composition balance seems to be one of the important factors in determining vascular stiffness [15,16]. Most of the previous studies conducted to determine which body composition has a greater effect on the relationship between body weight and bone density, that analyzed pre-and post-menopausal women, showed different results [33,34]. Notably, BMI is not an adequate indicator of the distribution of adipose tissue and is indistinguishable from muscle mass weight. It should be noted that a simple BMI can lead to analytical errors in patients with CKD and altered volume status. Although BMI has been reported to negatively affect bone microarchitectural integrity in the general population, including postmenopausal women [35,36], the relationship between BMI and TBS remains unclear [37].

Elderly and CKD patients are thought to have the same risk factors for a combination of diseases, such as osteoporosis and sarcopenia. Considering that muscles and bones have the same origin as mesenchymal cells, have similar neuroendocrine responses to loads, and communicate with each other, it may be necessary to examine them as a single unit [38]. Nonetheless, most of the previous studies have been conducted separately on bones or muscles, and new concepts in the category of osteosarcopenia have recently been introduced [39]. As shown in Table 1 and Table 2, the patient’s height appeared to be related to cAIx75. Therefore, we used LTI (kg/m^2^) standardized to height rather than just lean tissue mass (kg) for analysis. Osteopenia and sarcopenia may have accidentally occurred and progressed together due to the nature of CKD, but there are few studies evaluating the interaction with common clinical outcome parameters such as vascular stiffness at the same time. In this study, TBS and LTI were negatively related to cAIx75, respectively. Moreover, the negative B-coefficient for the interaction term was highlighted, which means that vascular stiffness tends to decrease more gradually as bone microarchitecture increases and when a larger value of lean tissue mass is given. Furthermore, the interaction effect between TBS and LTI was significantly noted only in women after correcting for age. Additionally, there was a significant interaction between these two variables after adjusting for age and sex only in the group with diabetes, not in the group without diabetes. Considering all of these results, the simultaneous assessment of bone and muscle mass loss, especially in the aforementioned groups, should be considered.

Although we showed significant interactions between bone microarchitectural integrity and muscle mass on vascular stiffness in advanced-stage CKD, we could not ascertain which biochemical laboratory parameter has a significant association with vascular stiffness. These results can be an obstacle in planning an efficient and effective prevention or treatment methods. Nevertheless, in contrast with our relatively homogeneous group, if patients with CKD in the early and late stages were included, a significant factor might have been found. Along with decreased kidney function, uremic toxins, hormonal changes, inflammation, and malnutrition more commonly affect muscle and bone metabolism. Malnutrition is one of the modifiable factors. In our study, the phase angle known as a nutritional marker was significantly associated with TBS (*r* = 0.297, *p* = 0.001) and LTI (*r* = 0.472, *p* < 0.001). The comparison of the mean of phase angle according to the three cAIx75 tertiles showed a marginal significance (*p* = 0.050). Protein energy wasting (PEW) common in CKD may be one of the causative or progressive factors of co-existence of osteopenia and sarcopenia. These unfavorable changes are likely to lead to poor clinical outcomes than each of these conditions alone. Malnutrition, particularly protein deficiency, contributes to the development of osteoporotic fractures by altering muscle function and reducing bone mass [40,41,42]. Therefore, nutritional support, especially protein supplementation with a well-controlled serum phosphate concentration, can play an important role in preventing or attenuating bone and muscle abnormalities in patients with advanced CKD and dialysis.

Some limitations of this study should be considered. This was a single-center study that included a relatively small number of patients. We did not measure the severity of VC at the same time using other evaluation tools such as quantitative computerized tomography. Furthermore, additional variables that may interfere with vascular stiffness may also exist, because our patients were accompanied by complicated combinations of PEW, increased uremic toxin, systemic inflammation, fluid imbalance, hemodynamic instability, and medications. These factors were not sufficiently considered in this analysis. Since not all stages of CKD patients were included and serial evaluations of the predictor, moderator, and dependent parameter were not performed over time, it is unlikely that our results can be generalized and applied to the patients of other earlier stages. Notably, the majority of patients with CKD comprise an extremely heterogeneous group with complex clinical features. Therefore, we acknowledge the possible influence of the complexity and diversity of disease on our results. Despite these limitations, the strengths of our study include that we performed the study on relatively homogenous patients with CKD5 who were free of non-traumatic bone fracture, and all aforementioned tests were simultaneously performed prior to the initiation of dialysis. To our best knowledge, this is the first study to have attempted such an analysis.

## 5. Conclusions

In CKD-MBD patients, the muscle mass index had an interaction in the association between VC-associated vascular stiffness and bone status. It is likely that malnutrition, a chronic problem in CKD patients, is closely related to osteosarcopenia and may contribute to deterioration of vascular stiffness. Therefore, our study suggests that the evaluation of body composition changes, bone health, and vascular stiffness needs to be performed simultaneously in patients with advanced-stage CKD.

## Figures and Tables

**Table 1:** Comparisons of demographics, bioimpedance parameters, serum chemistry, and bone status according to the tertile distribution of the augmentation index at 75.

Variables	Total (*n* = 117)	Augmentation Index at 75			*p*-Value
		Tertile 1 (*n* = 40)	Tertile 2 (*n* = 39)	Tertile 3 (*n* = 38)	
Augmentation index at 75, %	76.68 ± 14.37	62.03 ± 7.82	76.03 ± 3.23 ^a^	92.76 ± 8.61 ^b c^	<0.001
Age, years	59.38 ± 14.00	56.08 ± 13.65	59.49 ± 15.27	62.76 ± 12.44	0.107
Sex					
Male	63 (53.8%)	30 (47.6%)	23 (36.5%)	10 (15.9%)	<0.001
Female	54 (46.2%)	10 (18.5%)	16 (29.6%)	28 (51.9%)	
Diabetes					
Yes	75 (64.1%)	24 (32.0%)	28 (37.3%)	23 (30.7%)	0.471
No	42 (35.9%)	16 (38.1%)	11 (26.2%)	15 (35.7%)	
TBS, Lumbar spine	1.37 ± 0.11	1.41 ± 0.09	1.37 ± 0.11	1.32 ± 0.12 ^b^	0.001
FN-BMD, g/cm^2^	0.64 ± 0.14	0.68 ± 0.13	0.66 ± 0.13	0.59 ± 0.14 ^b^	0.010
FN-BMD T-score	−1.62 ± 1.16	−1.34 ± 1.02	−1.47 ± 1.13	−2.05 ± 1.24 ^b^	0.018
LS-BMD, g/cm^2^	0.96 ± 0.18	1.00 ± 0.17	0.98 ± 0.16	0.89 ± 0.19 ^b^	0.014
LS-BMD T-score	−0.51 ± 1.48	−0.16 ± 1.42	−0.35 ± 1.31	−1.03 ± 1.59 ^b^	0.023
cBMI, kg/m^2^	24.03 ± 4.15	23.50 ± 3.52	24.77 ± 4.41	23.83 ± 4.47	0.371
Height, cm	161.7 ± 8.61	165.1 ± 8.04	163.3 ± 8.51	156.4 ± 6.74 ^b c^	<0.001
LTI, kg/m^2^	12.28 ± 2.57	13.33 ± 2.37	11.96 ± 2.36 ^a^	11.49 ± 2.64 ^b^	0.004
FTI, kg/m^2^	11.57 ± 4.76	10.03 ± 4.07	12.62 ± 4.97 ^a^	12.11 ± 4.93	0.036
hs-CRP, mg/dL	0.71 ± 2.04	1.13 ± 3.22	0.60 ± 1.24	0.40 ± 0.57	0.272
iPTH, pg/mL	348.8 ± 272.2	339.9 ± 257.7	340.8 ±164.7	366.4 ±365.9	0.891
Vitamin D3, ng/mL	15.57 ± 10.11	17.09 ± 12.50	14.25 ± 7.45	15.34 ± 9.71	0.456
Total Cholesterol, mg/dL	139.03 ± 42.72	135.70 ± 51.38	137.05 ± 37.20	144.55 ± 38.34	0.622
Triglyceride, mg/dL	135.60 ± 67.35	129.50 ± 59.40	128.41 ± 58.68	149.39 ± 81.69	0.309
ALP, U/L	82.85 ± 37.74	76.55 ± 26.35	87.92 ± 37.33	84.26 ± 47.23	0.395
Calcium, mg/dL	7.65 ± 1.07	7.69 ± 1.02	7.60 ± 1.17	7.66 ± 1.03	0.931
Phosphate, mg/dL	6.06 ± 1.70	5.95 ± 1.77	6.02 ± 1.54	6.23 ± 1.81	0.756
eGFR, mL/min/1.73 m^2^	6.50 ± 2.33	6.70 ± 2.29	6.71 ± 2.35	6.08 ± 2.36	0.401

^a^ significant between tertile 1 and tertile 2, ^b^ significant between tertile 1 and tertile 3, ^c^ significant between tertile 2 and tertile 3. The cAIx75 tertiles 1, 2, and 3 correspond to <70%, 70–81%, and >81%, respectively. ALP, alkaline phosphatase; cBMI, corrected body mass index; eGFR, estimated glomerular filtration rate; FTI, fat tissue index; FN-BMD, femur neck bone mineral density; D.4.0tation index at 75ndex at 75 hs-CRP, high-sensitivity C-reactive protein; iPTH, intact parathyroid hormone; LTI, lean tissue index; LS-BMD, lumbar spine bone mineral density; TBS, trabecular bone score.

**Table 2:** Correlation between variables.

Variables	Augmentation Index at 75		Trabecular Bone Score, Lumbar Spine (L1–L4)		Lean Tissue Index	
	Correlation Coefficient	*p*-Value	Correlation Coefficient	*p*-Value	Correlation Coefficient	*p*-Value
Age, years	0.212	0.022	0.369	<0.001	−0.029	0.759
cBMI, kg/m^2^	−0.023	0.802	−0.305	0.001	0.055	0.556
Height, cm	−0.454	<0.001	0.574	<0.001	0.452	<0.001
hs-CRP, mg/dL	−0.084	0.372	0.129	0.128	0.142	0.096
iPTH, pg/mL	0.109	0.242	0.084	0.326	0.145	0.088
Vitamin D3, ng/mL	−0.098	0.296	−0.022	0.795	−0.020	0.812
Total Cholesterol, mg/dL	0.052	0.579	−0.010	0.029	0.048	0.572
Triglyceride, mg/dL	0.102	0.276	−0.135	0.114	−0.158	0.065
Alkaline phosphatase, U/L	0.035	0.707	−0.019	0.824	−0.024	0.778
Calcium, mg/dL	0.021	0.824	0.024	0.778	0.112	0.190
Phosphate, mg/dL	−0.020	0.831	0.133	0.119	0.145	0.019
eGFR, mL/min/1.73 m^2^	−0.053	0.569	0.023	0.788	0.050	0.561

cBMI, corrected body mass index; eGFR, estimated glomerular filtration rate; hs-CRP, high-sensitiity C-reactive protein; iPTH, intact parathyroid hormone.

**Table 3:** Results of hierarchical moderated regression analysis for testing moderating effects of the LTI for all patients.

Predictor	Augmentation Index at 75				
	Adjusted R^2^	B	SE	*Beta*	*p*-Value
*Demographics*	0.259				
Age		3.308	1.251	0.232	0.009
Sex		9.818	2.867	0.344	0.001
*Main effects*					
TBS	0.260	−1.310	1.303	−0.092	0.317
LTI	0.263	−2.228	1.379	−0.157	0.109
*Interaction*					
TBS × LTI	0.287	−2.461	1.120	−0.181	0.030

LTI, lean tissue index; TBS, trabecular bone score.

**Table 4:** Results of hierarchical moderated regression analyses for men and women.

Subgroup	Predictor	Augmentation Index at 75				
		Adjusted R^2^	B	SE	*Beta*	*p*-Value
Men	*Demographics*					
	Age	0.091	4.477	1.561	0.363	0.006
	*Main effects*					
	TBS	0.088	0.033	1.953	0.002	0.987
	LTI	0.087	−0.753	1.677	−0.057	0.655
	*Interaction*					
	TBS × LTI	0.111	−3.417	2.110	−0.229	0.111
Women	*Demographics*					
	Age	0.026	1.832	2.029	0.143	0.371
	*Main effects*					
	TBS	0.019	−3.089	2.116	−0.238	0.151
	LTI	0.014	−5.336	2.825	−0.311	0.065
	*Interaction*					
	TBS × LTI	0.073	−3.738	1.845	−0.347	0.048

LTI, lean tissue index; TBS, trabecular bone score.

**Table 5:** Results of hierarchical moderated regression analyses for patients with and without diabetes.

Subgroup	Predictor	Augmentation Index at 75				
		Adjusted R^2^	B	SE	*Beta*	*p*-Value
Patients without diabetes	*Demographics*	0.099				
	Age		3.119	2.400	0.244	0.202
	Sex		6.098	8.050	0.193	0.454
	*Main effects*					
	TBS	0.076	−1.787	3.423	−0.113	0.605
	LTI	0.053	−0.792	3.928	−0.050	0.841
	*Interaction*					
	TBS × LTI	0.069	−3.480	2.703	−0.215	0.206
Patients with diabetes	*Demographics*	0.378				
	Age		3.450	1.481	0.217	0.023
	Sex		11.486	2.899	0.421	<0.001
	*Main effects*					
	TBS	0.385	−1.709	1.297	−0.128	0.192
	LTI	0.404	−3.309	1.397	−0.249	0.021
	*Interaction*					
	TBS × LTI	0.431	−2.436	1.178	−0.196	0.042

LTI, lean tissue index; TBS, trabecular bone score.
